# Crossed-fused renal ectopia with renal calculi: Two case reports and a review of the literature

**DOI:** 10.1097/MD.0000000000018165

**Published:** 2019-11-27

**Authors:** Yalei Cao, Yinan Zhang, Weiting Kang, Ning Suo, Zilian Cui, Yuanman Luo, Xunbo Jin

**Affiliations:** Department of Urology, Shandong Provincial Hospital Affiliated to Shandong University, Jinan, China.

**Keywords:** calculi, crossed-fused renal ectopia, percutaneous nephrolithotomy, renal carcinoma

## Abstract

**Rationale:**

Crossed renal ectopia (CRE) is a rare congenital anomaly that is frequently associated with gastrointestinal, cardiovascular, genital and bone malformations. To the best of our knowledge, only 35 cases of crossed renal ectopia involving calculi and 30 cases of CRE associated with renal carcinoma have been reported to date.

**Patient concerns:**

Here, we present 2 cases of crossed renal ectopia. A 59-year-old woman with diabetes presented to our hospital with abdominal pain. The second patient was a 24-year-old woman who complained with abdominal pain with a duration of 1 day.

**Diagnoses:**

On the basis of abdominal ultrasonography, we suspected a solitary kidney both in the two patients. Combined with retrograde pyelography and 3D computed tomography, case 1 was diagnosed as an S-shaped right-to-left crossed-fused ectopic kidney with many stones in the left (normal) renal pelvis and case 2 was confirmed to have lump right-to-left crossed-fused renal ectopia with two 3-mm stones in the renal pelvis of the 2 kidneys.

**Interventions:**

Case 1 underwent percutaneous nephrolithotomy while case 2 refused to undergo surgery and underwent conservative treatment for pain relief.

**Outcomes:**

Two patients have been followed up and have no stones recurrence.

**Lessons:**

Crossed fused renal ectopia is easily misdiagnosed as a solitary kidney. CRE is so rare that the recognition of the disease needs to be improved and effective treatment should be taken timely. According to the two cases and literature review, minimally invasive surgery has become increasingly common to treat CRE with stones and carcinoma.

## 1 Introduction

Crossed renal ectopia is a rare congenital anomaly in which the kidneys are located on the same side, whereas the ureter of the ectopic kidney still descends to the normal location on the bladder. The anomaly consists of two types: crossed-unfused renal ectopia and crossed-fused renal ectopia, the latter of which is more common. The incidence of crossed-fused renal ectopia on autopsy and live birth has been reported to be approximately 1:7500 and 1:1000, respectively, with a male predominance.^[1–3]^ The symptoms of the anomaly are not obvious, and most individuals cannot be diagnosed until they undergo a medical examination. According to available published studies, the anomaly is associated with many malformations, including nephrolithiasis, ureteropelvic junction obstruction, renal tumors and cystic dysplasia.^[4–6]^ Hence, early diagnosis and effective therapeutic methods are beneficial. We searched PubMed from 1937 to 2018 with key words such as crossed fused ectopic kidney, crossed-fused renal ectopia, crossed renal ectopia, crossed renal ectopia calculi or stones, and crossed renal ectopia carcinoma or cancer. To date, only 35 cases of in crossed ectopic kidneys involving stones and 30 cases associated with renal carcinoma have been reported. The clinical data are presented in Tables 1 and 2. Although a few cases managed by surgery have been reported, there are no standard guidelines for treating crossed-fused ectopic kidneys with stones or renal carcinoma.

**Table 1:**
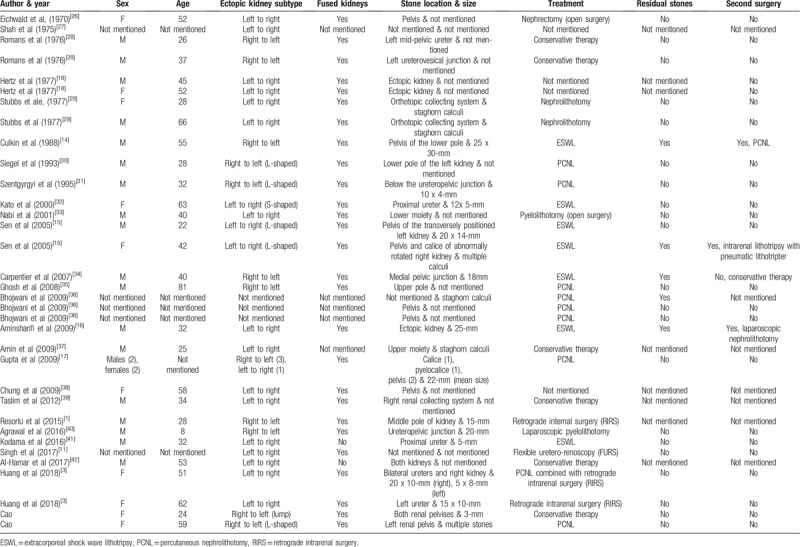
Review of reported crossed renal ectopia cases associated with stones.

**Table 2:**
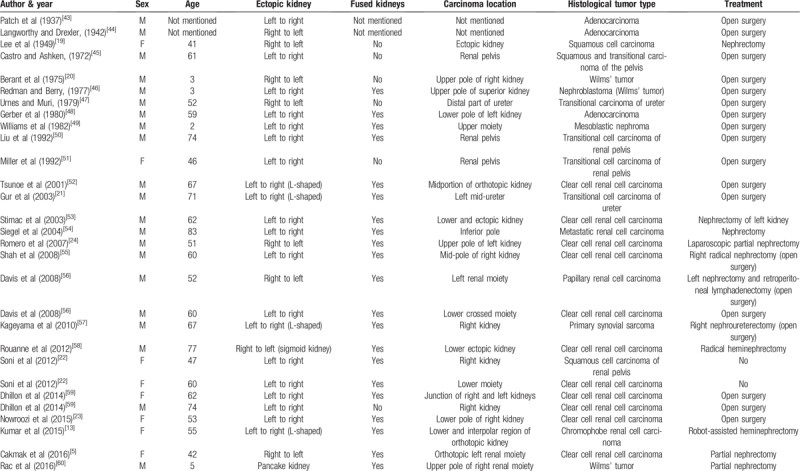
Review of reported cases of crossed renal ectopia associated with renal carcinoma.

Here, we report 2 cases of crossed-fused ectopic kidney with stones managed by percutaneous nephrolithotomy and medicine and review the available English literature.

## 2 Case report

### 2.1 Case 1

**Figure 1:**
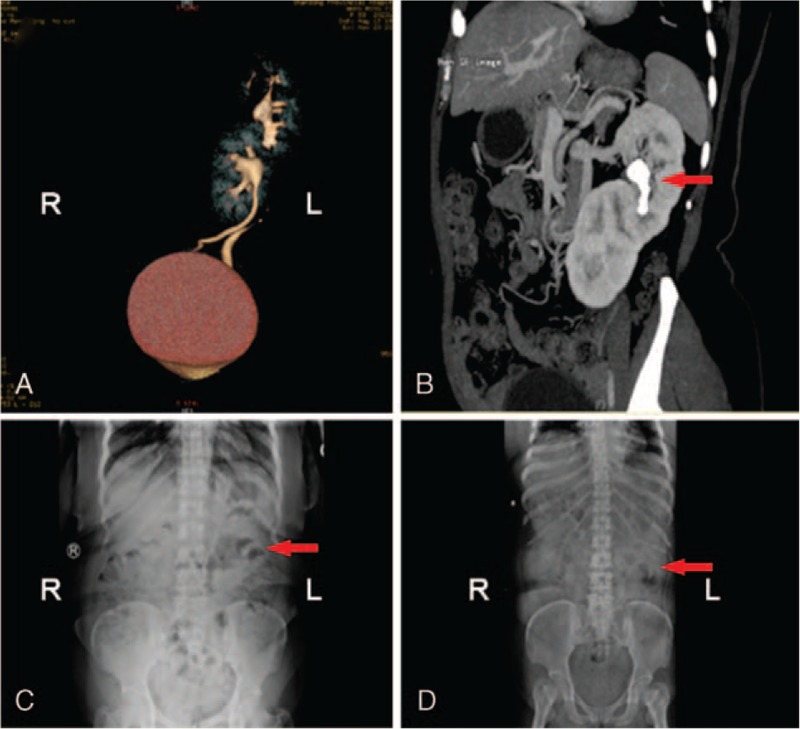
3D computed tomography and X-ray images of patient 1. (A) 3D computed tomography revealed S-shaped right-to-left crossed-fused renal ectopia. (B) CT demonstrated the vascular anomaly and calculi in the left renal pelvis (arrow). (C) Preoperative abdominal X-ray revealed stone shadows (arrow) in the left abdominal area. (D) Postoperative KUB showed no stone shadows.

A 59-year-old woman with diabetes presented to our hospital with abdominal pain. Her physical examination showed dull pain in the abdomen and no fever or flank pain on percussion. The laboratory findings revealed pyuria (white blood cell count in urine: 87.8 HPF). The renal function and other laboratory tests showed no abnormalities. Abdominal ultrasonography revealed the absence of the right kidney and a solitary left kidney with two ureters. For further examination, plain film of kidney-ureter-bladder (KUB), retrograde pyelography and 3D computed tomography were used to confirm the anomaly. 3D computed tomography showed an S-shaped right-to-left crossed-fused ectopic kidney with many stones in the left (normal) renal pelvis. (Fig. 1A) The crossed ectopic kidney was located inferior to the left kidney with fusion. Vascular anomalies were also found that the right renal artery was supplied by the anterior wall of the abdominal aorta, while the lower right renal vein passed up into the left renal vein. After considering the benefits and risks, the patient agreed to undergo PCNL owing to her rare anomaly and aberrant renal anatomy (Fig. 1B).

After the induction of general anesthesia, the patient was placed in the lithotomy position for inspection of the bladder and ureters using a rigid ureteroscope, and abnormities were found. Then, 2 5-F ureteral stents were placed in the left ureter and an 18-F catheter was placed before the patient was moved into the right lateral decubitus position. Under ultrasound guidance, the left renal pelvis was accessed, and the guide wire was placed in the tract. The stones were confirmed and visualized by the ureteroscope going through the tract. The biggest diameter of these stones was about 9-mm. After dilating the tract to 24-F, we combined the rigid nephroscope with a pneumatic lithotripter to fragment and eliminate the stones. Finally, a double-J tube was placed in the left ureter, and a 14-F nephrostomy tube was routinely placed in the tract after complete stone clearance. The procedure lasted 80 minutes and was successful without the need for blood transfusion or the occurrence of any complications. The patient underwent renal ultrasound before the 14-F nephrostomy tube was removed 6 days after the surgery. We compared the pre- and postoperative KUB findings to ensure complete stone clearance (Fig. 1C and D). After a year of follow-up, the patient stayed asymptomatic with no stone recurrence detected by abdominal ultrasonography.

### 2.2 Case 2

**Figure 2:**
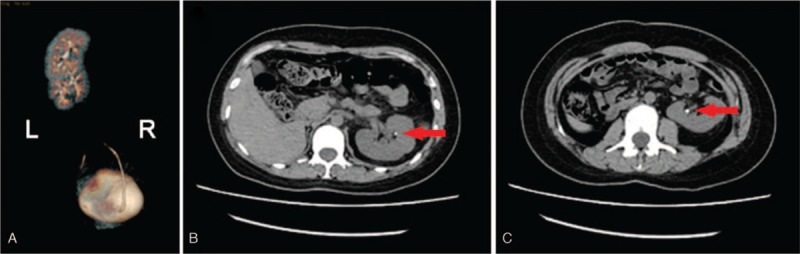
3D CT images of patient 2. (A) 3D CT image revealed lump right-to-left crossed-fused renal ectopia. (B, C) CT demonstrated two 3-mm stones in both renal pelvises.

In the second case, the patient was a 24-year-old woman. She presented with abdominal pain with a duration of 1 day. The physical examination showed acute pain in the left flank. The laboratory tests revealed pyuria and hematuria (white blood cell count in urine: 33.7 HPF; red blood cell count in urine: 258.4 HPF). Abdominal ultrasonography showed absence of right kidney on the right side. 3D computed tomography revealed right-to-left crossed renal ectopia and two 3-mm stones in the renal pelvis of the two kidneys. (Fig. 2) The ectopic kidney fused with the normal kidney and the ureter of the ectopic kidney descended and crossed the midline, just anterior to the fifth lumbar vertebra. The patient refused to undergo surgery and underwent conservative treatment for pain relief. At length, the stones were completely cleared without surgery, which was confirmed by abdominal ultrasonography. The symptoms of flank pain, pyuria and hematuria disappeared. She remained asymptomatic after 12 months of follow-up. The patient underwent abdominal ultrasonography during the follow-up because she refused the expense of the CT scan.

## 3 Discussion

The incidence of crossed-fused renal ectopia on autopsy has been reported to be approximately 1:7500, which is about 10 times higher than that of the unfused ectopia.^[1–3]^ In crossed-unfused renal ectopia, the ectopic kidney crosses the midline but the renal parenchyma of ectopic kidney does not fuse with that of normal kidney. The ureter of the ectopic kidney descends and crosses the midline and enters the urinary bladder at its normal position. In crossed-fused renal ectopia, the ectopic kidney crosses to the opposite side and fuses with the normal kidney. The ectopic kidney is usually located inferior to the normal kidney with fusion. Crossed-fused renal ectopia is a rare congenital abnormal development of the urinary system. There are 6 subtypes of the anomaly:

(1) inferior ectopia, in which the ectopic kidney lies inferior to the normal kidney;
(2) superior ectopia, in which the upper pole of the normal kidney fuses with the lower pole of the ectopic kidney;
(3) sigmoid or S-shaped;
(4) lump or pancake;
(5) L-shaped; and
(6) disk.^[7,8]^

Zhuo Yin et al, however, reported a new subtype of crossed-fused renal ectopia named the “Y” type, in which the ureters of the kidneys are fused.^[2]^ In our report, a 59-year-old woman presented with the S-shaped subtype and a 24-year-old woman presented with the lump subtype.

The diagnosis of crossed-fused renal ectopia can be confirmed by abdominal ultrasonography, KUB, retrograde pyelography and 3D computed tomography before surgery. First, ultrasonography can reveal the absent kidney. KUB and retrograde pyelography can help determine the size and location of any stones present. Attention should be paid to the 3D CT findings because the identification of essential vessels will allow access while avoiding bleeding and unnecessary mishaps.^[5]^ Vascular anomalies are always associated with crossed-fused renal ectopia. In patient 1 in the present study, the right renal artery was supplied by the anterior wall of the abdominal aorta, while the lower right renal vein passed up into the left renal vein, and the two veins then joined the inferior vena cava (Fig. 1B). Moreover, CRE may be associated with urinary abnormalities, such as urinary tract infection, renal calculi and ureteropelvic junction obstruction, principally due to mechanical reasons.^[1,9]^

This anomaly is so uncommon that there are no standard guidelines for treating associated stones or carcinoma. Minimally invasive measures have been increasingly reported to be helpful in managing this anomaly in association with nephrolithiasis, UPJO, and carcinoma, among others.^[3,10–13]^ After reviewing the available literature, we found 35 cases of crossed renal ectopia associated with stones. The female-to-male ratio was 10:21, and the ratio of the side of the ectopic kidney was 13:20 (left:right). The treatment methods included open surgery in 4 patients, conservative therapy in 5, extracorporeal shock wave lithotripsy (ESWL) in 7, percutaneous nephrolithotomy (PCNL) in 10, retrograde intrarenal surgery (RIRS) in 4 and laparoscopic pyelolithotomy in 1. However, the treatment method in 5 cases was not mentioned, and in 3 patients, ESWL failed, and a second surgery was required.^[1,14–18]^ In our report, patient 1 was completely cleared of stones with PCNL, and patient 2 underwent conservative treatment. After reviewing available studies in the literature thoroughly, we found 30 cases of renal cancer in CRE.^[5,19–22]^ We also found that CRE associated with renal cancer mainly occurs in adults, most commonly as clear cell renal cell carcinoma.^[23]^ Only 4 cases of renal carcinoma in CRE in children have been reported, 3 of which were cases of Wilms’ tumor (Table 2).

The choice of treatment in CRE with stones depends on the vascular anatomy and the size of the stone. As far as we are concerned, patients with small stones without hydronephrosis, such as patient 2, may be treated with conservative therapy. However, those with large calculi and hydronephrosis, such as patient 1, should be treated with surgery to achieve stone clearance. Watchful waiting and close follow-up should be recommended for patients without symptoms because they may be asymptomatic throughout their life. The treatment methods for stones in CRE include open surgery, ESWL, PCNL, RIRS and laparoscopic surgery. With the development of surgical instruments, minimally invasive surgery has become increasingly common and feasible, while the selection of open surgery is gradually decreasing (Table 1). ESWL is an alternative treatment, although it was reported to have failed in 3 patients who had residual stones and required a second surgery.^[14–16]^ Sen et al reported the use of ESWL to treat 2 patients with an L-shaped renal anomaly with stones. The first patient was free of stones after three sessions, while the second patient was treated with retrograde intrarenal lithotripsy after ESWL failed.^[15]^ Aminsharifi et al reported the use of laparoscopic nephrolithotomy to treat a patient with a 25-mm opaque renal stone in left-to-right crossed-fused renal ectopia who had undergone two sessions of ESWL that failed.^[16]^

Percutaneous nephrolithotomy (PCNL) is another alternative, especially in patients with a large stone burden; however, the risks of bleeding and gastrointestinal injury may increase due to the aberrant vascular supply and the location of the ectopic kidney. Gupta et al reported the use of PCNL to treat 46 patients (52 renal units) with abnormal kidneys, including 4 patients with crossed-fused ectopic kidneys. The mean size of the stones was 22 mm, and the mean operating time was 80 minutes (range, 70–100).^[17]^ Huang et al reported 2 cases of crossed-fused renal ectopia, one with 20-mm stones and one with 15-mm stones. One of the patients underwent PCNL and achieved total stone clearance.^[3]^

Additionally, to the best of our knowledge, Resorlu et al reported the first use of RIRS in a patient with crossed-fused renal ectopia with a 15-mm stone, which confirmed that RIRS is a safe and feasible minimally invasive choice for application in cases of this anomaly.^[1]^

Concerning the reports of renal cancer in crossed renal ectopia, clear cell renal cell carcinoma was the most common, and the main surgical method for treatment was open surgery (Table 2). Romero et al were the first to apply laparoscopic heminephrectomy to treat a patient with clear cell renal cell carcinoma associated with right-to-left crossed-fused renal ectopia.^[24]^ Kumar et al reported the first use of robot-assisted heminephrectomy to treat chromophobe renal cell carcinoma in a case of L-shaped renal ectopia. According to the location, size and clinical stage of the renal tumor, radical or partial nephrectomy should be performed as soon as possible to preserve the functional and uninvolved kidney. Crossed-fused ectopic kidneys can even be utilized as donor organs for transplantation.^[25]^

## 4 Conclusion

Because of the rarity of crossed renal ectopia, detailed and accurate preoperative examinations and assessments must be emphasized for the diagnosis of the anomaly. The choice of treatment in CRE with stones relies on the vascular anatomy, the size of the stones, the extent of hydronephrosis and the experience of the urologist. Based on the cases provided from the literature, minimally invasive surgery has become increasingly common to treat CRE with stones and carcinoma. Urologists should choose safe, sufficient and feasible methods to cure CRE with abnormalities involving stones, carcinoma and other malformations.

## Author contributions

**Conceptualization:** Yalei Cao, Yinan Zhang, Weiting Kang, Yuanman Luo, Xunbo Jin.

**Data curation:** Yalei Cao, Yinan Zhang, Weiting Kang, Ning Suo, Zilian Cui, Yuanman Luo.

**Funding acquisition:** Zilian Cui, Xunbo Jin.

**Methodology:** Weiting Kang, Ning Suo, Yuanman Luo, Xunbo Jin.

**Supervision:** Yinan Zhang, Weiting Kang, Ning Suo, Zilian Cui.

**Visualization:** Ning Suo, Zilian Cui, Yuanman Luo, Xunbo Jin.

**Writing – original draft:** Yalei Cao.

**Writing – review & editing:** Yalei Cao.

Yalei Cao orcid: 0000-0002-4197-5547.
